# Interpersonal predictors of loneliness in Japanese children: variable- and person-centered approaches

**DOI:** 10.1186/s40359-022-00853-1

**Published:** 2022-06-29

**Authors:** Takuma Nishimura, Tatsuya Murakami, Shigeo Sakurai

**Affiliations:** 1 Faculty of Education, Fukuyama City University, Hiroshima Fukuyama, Japan; 2 Faculty of Health and Sports Science, Juntendo University, Inzai, Chiba Japan; 3 Graduate School of Comprehensive Human Sciences, University of Tsukuba, Tsukuba, Ibaraki Japan

**Keywords:** Children, Hierarchical linear modeling, Loneliness, Longitudinal study, Mover-stayer latent transition analysis

## Abstract

**Background:**

Loneliness in children has been a major topic of interest in both clinical and developmental psychology. Further studies to investigate predictors of loneliness are needed for educational practices.

**Methods:**

A total of 1088 late elementary school-aged children (48.81% boys, M_age_ = 10.35) participated in a 1-year longitudinal survey. We used hierarchical linear modeling and mover-stayer latent transition analysis.

**Discussion:**

Findings from the variable- and person- centered approaches suggested that less positive peer relations, higher victimization, and higher relational aggression are predictors of higher future loneliness. Meanwhile, both approaches did not reach an agreement concerning predictors to reduce loneliness. This result highlighted a utility of a combined approach and sounded an alarm for overreliance on the variable-centered approach dominating child research.

**Conclusion:**

To protect young children from loneliness, it will be more beneficial to prevent the development of loneliness rather than to apply interventions to reduce loneliness once established. Preventive practices need to be implemented to protect children from loneliness.

**Supplementary Information:**

The online version contains supplementary material available at 10.1186/s40359-022-00853-1.

## Background

Feelings of loneliness in children have been a major topic of interest in clinical and developmental psychology [13]. Considerable research has revealed that loneliness is related to numerous problems of clinical relevance and risks including depression, anxiety, social withdrawal and suicide ideation [4, 11, 15, 34]. Additionally, lonely individuals tend to suffer from a cognitive bias toward threatening social stimuli, and easy access to the experience of loneliness [39]. This means that an experience of loneliness makes the individual more sensitive to loneliness and makes memories of maladaptive experiences more readily accessible.

According to van Dulmen and Goossens [41], 3–14% of children or adolescents experienced stable high or chronic loneliness; this means that they were exposed to loneliness longitudinally. Therefore, research involving its predictors is significant in terms of elucidating how to prevent loneliness in children. The primary interest of the present study was to examine interpersonal predictors of loneliness in late elementary school-aged children through a combination of variable- and person- centered approaches.

### Predictors of loneliness in children

Loneliness is a subjective experience and an emotional tendency described as sadness or pain, which is caused by an absence of connection with others [31]. Abundant research to date has focused on interpersonal experiences as predictors of loneliness because the primary trigger in children of loneliness is thought to be a lack of or difficulties in peer relations [1, 2]. Under this assumption, which provides a rationale for research regarding predictors of loneliness, poor peer relations (such as lower peer acceptance and social competence) and peer victimization have been examined whether they are valid predictors of loneliness. For instance, Ladd, Kochenderfer, and Coleman [21] reported that a significant proportion of variance in loneliness accounted by positive peer relations was of medium effect size in both cross-sectional (*R*^2^ = 0.19) and longitudinal studies (*R*^2^ = 0.13) and that victimization also predicted changes in loneliness longitudinally. Ladd and Troop-Gordon [22] conducted a 5-year longitudinal study and found that chronic friendlessness and rejection accounted for a proportion of the variance in loneliness. Further, other studies using sociometric methods to obtain group members’ ratings or nominations of liked or disliked others showed that victimized children (i.e., those rejected) reported high loneliness compared to the accepted or liked children [6, 26].

Aggression, which may be classified as overt aggression (e.g., punching, kicking, threatening) and relational aggression (e.g., excluding, manipulating others’ relationships, spreading rumors [7]), which could push others away, resulting in rejection by peers because aggressive behaviors are negatively viewed [10] and unacceptable in peer relation [5, 30]. Such experiences made children easily feel lonely. In this way, aggression has been hypothesized to produce feelings of loneliness in a series of loneliness studies for children. However, as a potential interpersonal predictor of loneliness, the predictive role of aggression remains undefined. Prinstein, Boergers, and Vernberg’s [32] cross-sectional study showed no overall relation between the two types of aggression and loneliness. While Putallaz et al. [33] reported a positive relation between overt aggression and loneliness, no relation was shown between relational aggression and loneliness. Thus, further investigation is needed.

The above review shows that loneliness in children is deeply related to their interpersonal experience. Although many other potential antecedents such as friendship quality, social network, and individual characteristics has been explored [8], the present study focused on the role of key interpersonal predictors: positive peer relations, victimization, and the two types of aggression (overt and relational aggression). In addition, the theory of loneliness has given much attention to the causes of loneliness such as poor peer relations, higher victimization, and higher aggression. However, researchers of child loneliness should also focus on positive aspects of interpersonal experiences as resilience such as healthy peer relations, lower victimization, and lower aggression. As described below, by utilizing analysis of person-centered approach, we can more precisely investigate both potentials of interpersonal relations for child loneliness.

### Person-centered approach

Though several variable-centered approaches have dominated this research area, incorporation of person-centered approaches for interpretation has been recommended in developmental psychopathology [39]. The person-centered approaches focus on the identification of groups that share specific attributes or individual differences in developmental patterns [24]. As evident in Kochenderfer-Ladd and Wardrop’s [18] study on victimization trajectories, one of the strengths of person-centered approaches is to provide more detailed insights into relations. Besides adopting a variable-centered view, in which a clear connection exists between victimization and loneliness, they adopted a person-centered view by focusing on trajectory patterns. A person-centered view illustrated that while children moving toward victimization status tended to experience greater loneliness, they did not necessarily become less lonely when moving away from victimization status.

In the first place, previous research on children’s loneliness focusing on predictors seems to have relied on a variable-centered approach with not much attention given to the potential over-interpretation of covariates. For example, when a negative longitudinal relation is found between an independent variable (i.e., potential predictors) and loneliness, researchers can interpret the relation as indicating both that a lower score on the independent variable is predictive of increased feelings of loneliness and that a higher score on the independent variable is predictive of decreased feelings of loneliness. However, this interpretation is not always applicable because a mere correlation does not necessarily mean that both conditions are simultaneously satisfied; that is, only one condition is well established and then the negative relation emerges. Therefore, in addressing the issue of potential over-interpretation of covariates, a person-centered approach is useful.

To investigate the predictors of loneliness from a person-centered approach, mover-stayer latent trajectory analysis (mover-stayer LTA) is applicable. The mover-stayer LTA explores transitional patterns between latent profiles across time. It differs from typical LTA in being able to identify participants whose latent profiles change (i.e., movers) or remain the same (i.e., stayers) during the study period. Additionally, we can confirm whether the results of a variable-centered approach are in the same direction by applying mover-stayer LTA by comparing the scores of potential predictors at specific time points. For example, if positive peer relations are strongly related to reduced loneliness (variable-centered view), individuals with latent profiles characterized by decreasing loneliness should have higher scores for positive peer relations during the period of the assessments (person-centered view).

The other advantage of mover-stayer LTA is to widely cover potential patterns of trajectories. Here, we reviewed seven empirical research that identified loneliness trajectories in children [3, 12, 14, 20, 35, 38, 42], revealing specific subpopulation of trajectory patterns such as low levels of loneliness, decreasing, increasing, and chronically high patterns. However, sub-groups of loneliness trajectories were not consistent among the previous studies; for example, almost all studies reported a trajectory of individuals with decreasing loneliness except for Benner [3]. Moreover, five studies reported an increasing trajectory, but Harris et al. [12] and Ladd and Ettekal [20] did not report the increasing pattern. As reviewed, there were several types of loneliness trajectories; however, the largest percentage of children belonged to a low stable pattern among reported trajectories in each study. Although there was some variation among the studies, approximately 40–80% of children were classified into a pattern of low or no loneliness. Specifically, four studies [3, 12, 14, 42] reported that more than half of children in each sample are categorized low stable pattern of loneliness. In contrast, around 10% of the children were exposed to chronic loneliness and approximately 10–20% belonged to increasing or decreasing patterns of loneliness. Thus, there is an interindividual variation in the developmental changes in children’s sense of loneliness. The mover-stayer LTA can help to detect the diversity of the differentiation.

### Overview of the present study

The purpose of the present study was to identify predictors of loneliness through variable- and person-centered approaches. First, using hierarchical linear modeling (HLM) as the variable-centered approach, we investigated the effect of positive peer relations, victimization, and the two types of aggression on loneliness. Second, using mover-stayer LTA as the person-centered approach, we tested the replication of the results by HLM. The hypothesis regarding the predictors of loneliness in HLM was that victimization and relational aggression would be positively correlated, and positive peer relations would be negatively correlated with loneliness. In addition, the results of the mover-stayer LTA might support the direction of the outcome of the HLM.

## Method

### Study design

This study involved a 1-year longitudinal design with three measurement points, with about half a year interval. We conducted the baseline assessment (Time 0: T0) for loneliness between January and February, the second assessment (Time 1: T1) for loneliness and the potential predictors (i.e., positive peer relations, victimization, and two types of aggression) between June and July, and the third assessment (Time 2: T2) for loneliness a year after baseline between January and February. These interpersonal predictors were assessed at only T1 to match the results of HLM and mover-stayer LTA.

### Participants

A total of 1088 elementary school students in the 4th and 5th grade (531 boys and 557 girls; *M*_age_ = 10.35, age range was 9–11) from eight public schools (37 classes) in Japan participated in the survey. The number of participants with complete data was 1054 at T0, 1046 at T1, 1071 at T2, and 1029 throughout the three measurement points. All participants were Japanese from the lower to upper middle-class socioeconomic status. The sample was highly homogeneous in terms of ethnic and cultural back ground based on their demographics.

### Measures

#### Loneliness (children’s report)

We used the Five-item Loneliness Scale for Children (Five-LSC; [28]). All five items use direct expressions to assess loneliness (Item 1: “Do you feel that you are alone?”, Item 2: “Do you think that there is nobody to play with?”, Item 3: “Do you feel that you are left behind by people around you?”, Item 4: “Do you think that no one would help you if you were in trouble?”, Item 5: “Do you feel lonely?”). According to Nishimura et al. [28], the Five-LSC has demonstrated sufficient validity based on teachers’ observational assessment: the difference in the score of loneliness between lonely students nominated by teachers and non-nominated was significant (Cohen’s *d* = 0.60). Also, loneliness assessed by the Five LSC showed a negative correlation with social competence and social skills, but a positive correlation with withdrawn behaviors. These measures were developed for use in Japan and well validated in that context. The rating was a 4-point Likert scale ranging from 1 (strongly disagree) to 4 (strongly agree). We computed the arithmetic mean of the five items with higher scores reflecting higher loneliness. Participants responded to the Five-LSC at the three measurement points. Because of the underestimations for internal consistency of Cronbach’s alpha [9], we used McDonald’s omega (0.91, 0.93, and 0.94 at T0, T1, and T2, respectively).

#### Positive peer relations (children’s report)

We assessed positive peer relations with a three-item scale (e.g., “Are peers kind to you”) that was standardized and published by the Toshobunka Company in Japan. The scale was developed for use in Japan and has established clinical validity with low scores predicting peer victimization [17] and higher loneliness [29]. The rating is a 4-point ranging from 1 (strongly disagree) to 4 (strongly agree). We computed the arithmetic mean of all three items with higher scores reflecting more positive peer relations. In this study, McDonald’s omega of this scale was 0.83.

#### Victimization (children’s report)

We used a six-item scale of victimization standardized and published by the Tosyobunka Company in Japan. This scale was developed for use in Japan and has established high clinical and prospective validity, with high scores predicting non-attendance and being bullied at school [17]. The rating is a 4-point scale ranging from 1 (strongly disagree) to 4 (strongly agree). We used the arithmetic mean of all six items with higher scores reflecting higher victimization. In this study, McDonald’s omega of this scale was 0.89.

#### Relational and overt aggression (teachers’ report)

We used a teacher-report measure to assess children’s relational and overt aggression in reference to Click and Grotpeter [7]. This measure has seven items, four of which assess relational aggression (e.g., trying to exclude others from their group), and three items assess overt aggression (e.g., hits, pushes others) and content validity is established [7]. Items are rated on a five-point scale, ranging from 1 (never) to 5 (always). We used the arithmetic mean with higher scores reflecting higher aggression. Thirty-seven homeroom teachers rated items for each student in their homeroom. In this study, McDonald’s omega was 0.89 for relational aggression and 0.90 for overt aggression.

### Procedure

Homeroom teachers administered all questionnaire surveys in their class. The top sheet of the questionnaire contained the following statements: (a) the survey was not related to school grade evaluations, (b) the privacy of those taking the survey would be protected, and (c) participants were guaranteed the freedom to withdraw from participation. Participants provided their demographics data such as grade, class, sex, and student number on the last page of the questionnaire, which allowed us to link data from different time points and the teacher assessments.

This research project involved collaboration with eight public schools. We contributed to each school’s educational practice by providing feedback data regarding homeroom management and identifying students with a high risk of school maladjustment. The schools explained this project to parents, who provided informed consent on behalf of their children and obtained the agreement in this research. Also, participants were informed that they had the full freedom to participate or withdraw at any time of their participation by written consent. The institutional research board (IRB) at the authors’ university approved the procedures. All methods were performed in accordance with the guidelines.

### Statistical analysis

All analyses were performed using R 3.1.0 and Mplus ver. 7.11 [27]. We used HLM to examine the predictors of loneliness, because children were nested within classrooms. In HLM, variance in individual-level outcomes is partitioned into a child (within-level) and classroom (between-level) variance. Thus, we first calculated the intra class coefficient (ICC) to assess the percentage of the between-level variance in loneliness explained. The variance at the between-level was 0.02; although this variance was small, we applied HLM to avoid a type-1 error [19]. Furthermore, because of the nature of classrooms managed by homeroom teachers, we expected that between-level variances would moderate the connection between loneliness at T1 and T2. Thus, we were also interested in analyzing cross-level interactions. For ease of interpretation, between-level variables were centered at the grand mean in the analysis.

Using person-centered approaches, we first run latent profile analysis (LPA) and then mover-stayer LTA. LPA examines the number of underlying subgroups of participants with similar patterns based on latent factor scores. For determining the number of profiles which had the best fit to the hypothesized model, we referred to Akaike’s information criterion (AIC), Bayesian information criterion (BIC), adjusted BIC (aBIC), and the result of the bootstrap likelihood ratio test (BLRT). Models with relatively lower AIC, BIC, and aBIC values fit better than those with higher values. The BLRT compares the improvement in fit between neighboring class models. The null hypothesis is that the k profile solution fits better than a solution with k-1 profiles. A significant *p* value indicates the superiority of the k profile model compared to the k-1 model. Next, we employed to mover-stayer LTA to examine the transition of participants between latent profiles at T0 and T2. A mover-stayer LTA uses second-order latent class variables that classify individuals into “movers” and “stayers”. We reported the Mplus syntax of the mover-stayer LTA in Appendix 1 as Additional file 1. Then, we estimated the relation between the transition status identified by mover-stayer LTA and each score of the predictors: positive peer relations, victimization, relational and overt aggression at T1. Finally, we addressed missing data by using the full information maximum likelihood method. We checked the pattern of missing data and comparison of loneliness score of T2 between children with non-missing and missing at T0 and T1 and ascertained that the effect sizes of differences were small; therefore, we assumed that the data were missing at random.

## Results

### Preliminary analyses

Table 1 presents the means, standard deviations, and correlations among all variables. We observed moderately positive correlations between loneliness each time point (*r* = 0.44 to 0.62, *p *< .001). Positive peer relations were significantly negatively correlated with loneliness, while victimization was significantly positively correlated with loneliness. Neither type of aggression, except for relational aggression at T1, showed any correlation with loneliness.

**Table 1:** Means, standard deviations, 95% confidence intervals and correlations among the variables

	*M*	*SD*	95% CI	2	3	4	5	6	7
1. Loneliness (T0: baseline)	1.38	0.62	[1.34, 1.42]	.55***	− .36***	.43***	.02	.01	.44***
2. Loneliness (T1)	1.36	0.60	[1.32, 1.39]		− .50***	.65***	.06	.04	.62***
3. Positive peer relations (T1)	3.33	0.57	[3.30, 3.37]			− .49***	− .04	− .06	− .42***
4. Victimization (T1)	1.51	0.57	[1.47, 1.54]				.04	.06	.50***
5. Relational aggression (T1)	1.40	0.59	[1.37, 1.44]					.47***	.12***
6. Overt aggression (T1)	1.15	0.40	[1.13, 1.18]						.06
7. Loneliness (T2)	1.41	0.66	[1.37, 1.45]						

T0 = Time 0, T1 = Time 1, T2 = Time 2. CI = confidence interval

***p < .001

### Predictors of loneliness: hierarchical linear modeling

Table 2 presents the results of the HLM with a random intercept and slope model. After controlling for demographics and baseline variables, we found that loneliness at T1, victimization and relational aggression were positively correlated with loneliness at T2. Positive peer relations were negatively correlated with loneliness at T2. Overt aggression was not associated with loneliness at T2. Focusing on cross-level interactions, between-level victimization was positively correlated with the relation between loneliness at T1 and T2.

**Table 2:** The results of hierarchical liner modeling for loneliness at time 2

Variables	γ	*SE*	*p*	95% CI
*Within level*				
(Demographics and baseline variables)				
Gender	− 0.08	0.03	.010	[− 0.14, − 0.02]
Age	− 0.01	0.03	.749	[− 0.06, 0.05]
Loneliness (T0: baseline)	0.11	0.03	< .001	[0.05, 0.17]
(T1 variables)				
Loneliness	0.46	0.04	< .001	[0.36, 0.53]
Positive peer relations	− 0.13	0.03	< .001	[− 0.20, − 0.07]
Victimization	0.11	0.04	.002	[0.04, 0.19]
Relational aggression	0.09	0.04	.010	[0.02, 0.16]
Overt aggression	0.03	0.05	.719	[− 0.06, 0.13]
Cross-level interaction				
Positive peer relations with loneliness (T1)	0.42	0.32	.192	[− 0.21, 1.04]
Victimization with loneliness (T1)	0.82	0.35	.020	[0.13, 1.50]
Relational aggression with loneliness (T1)	− 0.09	0.08	.915	[− 0.42, 0.25]
Overt aggression with loneliness (T1)	− 0.11	0.33	.740	[− 0.74, 0.53]

T0 = Time 0, T1 = Time 1, CI = confidence interval

### Predictors of loneliness: mover-stayer LTA

We first investigated the number of profiles that best fit the data at T0 and T2. Although we first hypothesized that the number of profiles would be more than four, uninterpretable profiles emerged. Therefore, we compared the models from one- to three-profiles. Table 3 shows comparisons of the latent profiles. At both measurement points, AIC, BIC, and aBIC of the three-profile model were the smallest among the models. We found a significant difference among the models via the BLRT. Therefore, we performed the mover-stayer LTA using the three-profile model for exploring the transitions between these profiles across measurement points.

**Table 3:** The summaries of model comparison of latent profile models at both T0 and T2

Model	*df*	AIC	BIC	aBIC	∆LR	*p*
T0: Baseline						
One-profile model	10	12,103.91	12,153.72	12,121.96		
Two-profile model	16	9253.27	9332.96	9282.14	2862.65	< .001
Three-profile model	22	7741.47	7851.34	7781.47	1523.51	< .001
T2						
One-profile model	10	12,155.41	12,205.17	12,173.41		
Two-profile model	16	8615.42	8695.04	8644.22	3551.99	< .001
Three-profile model	22	7141.72	7251.20	7181.33	1485.69	< .001

AIC = Akaike’s information criterion. BIC = Bayesian information criterion. aBIC = sample size adjusted BIC. ∆LR means difference of likelihood ratio between neighboring class models (i.e., comparing k − 1 and the k class models)

**Fig. 1:**
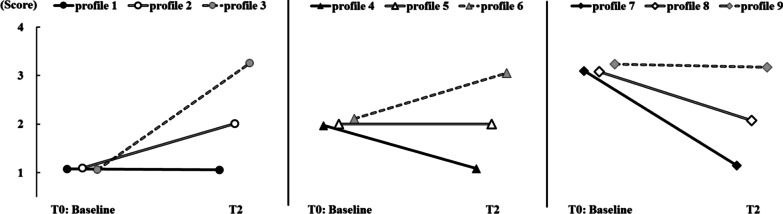
All transition patterns of mover-stayer LTA

Table 4 presents the characteristics of each profile. Profile 1 was the “low group” as characterized by low scores of loneliness (1.03 to 1.10). In total, 812 students at T0 and 792 students at T2 belonged to profile 1. Profile 2 was the “relatively low group” as characterized by relatively low scores of loneliness (1.93 to 2.09); overall, 210 students at T0 and 215 students at T2 belonged to profile 2. Finally, the “high group” under profile 3 was characterized by high scores of loneliness (2.95 to 3.35) in which 66 students at T0 and 81 students at T2. Table 5 and Fig. 1 present the trajectory patterns. Movers were those who transitioned from one profile to another across time. Stayers were those who remained in the same profile. Overall, 768 students (70.59%) were classified as stayers (i.e., patterns 1, 5, and 9) and 219 students (29.41%) as movers including 175 increasers (patterns 2, 3, and 6) and 145 decreasers (patterns 4, 7, and 8). The quality of classification of mover-stayer LTA was acceptable (entropy = 0.83). Table 6 summarizes the loneliness trajectories found in previous studies and their correspondence with the findings of the current study.

**Table 4:** Unstandardized means, standard errors, and 95% confidence intervals at each latent profile

	1. Low group			2. Relatively low group			3. High group		
	*M*	*SE*	95% CI	*M*	*SE*	95% CI	*M*	*SE*	95% CI
Item 1	1.07	0.01	[1.05, 1.10]	2.09	0.04	[2.01, 2.17]	3.32	0.06	[3.21, 3.43]
Item 2	1.08	0.01	[1.05, 1.10]	1.93	0.04	[1.85, 2.00]	2.95	0.08	[2.80, 3.10]
Item 3	1.03	0.01	[1.01, 1.04]	2.04	0.04	[1.97, 2.10]	3.35	0.05	[3.25, 3.46]
Item 4	1.10	0.01	[1.08, 1.03]	1.98	0.04	[1.90, 2.06]	2.98	0.08	[2.82, 3.13]
Item 5	1.08	0.01	[1.06, 1.00]	2.02	0.05	[1.94, 2.11]	3.16	0.08	[3.00, 3.31]
	Frequency (%)								
T0: Baseline	812 (74.63)			210 (19.30)			66 (6.07)		
T2	792 (72.79)			215 (19.76)			81 (7.45)		

CI = confidence interval

**Table 5:** Frequency of each mover-stayer latent transition pattern

Latent profile			Frequecny	%	Change of category
No	T0: baseline	T2			
1	Low group	Low group	671^a^	61.67	Stayer
2		Relateively low group	117^b^	10.75	Increaser
3		High group	24^b^	2.21	Increaser
4	Relateively low group	Low group	102^b^	9.38	Decreaser
5		Relateively low group	74^a^	6.80	Stayer
6		High group	34^b^	3.13	Increaser
7	High group	Low group	19^b^	1.75	Decreaser
8		Relateively low group	24^b^	2.21	Decreaser
9		High group	23^a^	2.11	Stayer

^a^"stayers": those participants who were classified into the same latent profile in T0 (baseline) and T2

^b^"movers": those participants who were classified into the different latent profile from T0 (baseline) and T2

**Table 6:** Ovrerview of loneliness trajectories and inclusiveness of mover-stayer LTA

	Profiles identified by mover-stayer LTA									Result
	1	2	3	4	5	6	7	8	9	
Benner [3]										
Chronically high (11%)									〇	Covered
Low-steady (78%)	〇									Covered
Low-increasing (11%)		〇								Covered
Harris et al. [12]										
Relative high, reducing loneliness (48%)							△	△		Partially covered
Low, stable loneliness (52%)	△				△					Partially covered
Jobe-Shields et al. [14]										
Stable low (66%)	〇									Covered
Increasing (22%)						〇				Covered
Decreasing (12%)							〇			Covered
Ladd and Ettekal [20]										
No loneliness (18.9%)	〇									Covered
Low loneliness (19.5%)	△				△					Partially covered
Moderate loneliness (41.6%)					〇					Covered
Declining loneliness (6.2%)				〇						Covered
Chronic loneliness (13.7%)									〇	Covered
Qualter et al. [35]										
Stable high lonely (22%)									〇	Covered
Moderate increasers (18%)						〇				Covered
Moderate decliners (23%)				〇						Covered
Low stable lonely (37%)	〇									Covered
Schinka et al. [38]										
Chronic (4.1%)									〇	Covered
Moderate increasing (31.6%)						△				Partially covered
High increasing (4.5%)			〇							Covered
Stable low (49.1%)	〇									Covered
Decreasing (10.7%)							〇			Covered
Vanhalst et al. [42]										
Chronically high (3%)									〇	Covered
High decreasing (8%)								〇		Covered
Moderate decreasing (9%)				〇						Covered
Low increasing (18%)		△								Partially covered
Stable low (63%)	〇									Covered

LTA = latent transition analysis, 〇 = match, △ = partially match

Next, we estimated factor scores of positive peer relations, victimization, and relational and overt aggression in order to examine the effects of children’s interpersonal experiences at T1 on these trajectories of loneliness. Table 7 presents the factor scores of positive peer relations and victimization. Table 8 presents scores for the two types of aggression for each profile along with the results of one-sample t-tests and their effect size (i.e., Cohen’s *d*). These factor scores represent distal means (from zero); the p value is associated with the null hypothesis (the mean is equal to zero). Overall, several statistically significant effects were found for positive peer relations, victimization, and relational aggression, but not for overt aggression. The stayer in pattern 1 was associated with higher peer relations, lower victimization, and lower relational aggression. Meanwhile, other profiles of the stayer (i.e., patterns 5 and 9) were associated with lower positive peer relations and higher victimization but had no relation with either type of aggression. As for the increasers (i.e., patterns 2, 3, and 6), they were associated with lower positive peer relations, higher victimization (chance level in profile 2), and relational aggression. As for the decreasers, those in patterns 7 and 8 were associated with higher victimization, while pattern 8 was also negatively correlated with positive peer relations. No significant effects were found for pattern 4. Surprisingly, although they were decreasers, their factor scores of positive peer relations and victimization were not positive and negative, respectively.

**Table 7:** Results of one sample t-test for factor scores of positive peer relations and victimization at each profile

Profiles		Positive peer relations						Victimization					
No.	Patterns	*M*	*SD*	95% CI	*t*	*p*	*d*	*M*	*SD*	95% CI	*t*	*p*	*d*
1	L–L	0.18	0.39	[0.15, 0.21]	11.63	< .001	0.45	− 0.21	0.38	[− 0.24, − 0.18]	− 14.16	< .001	0.55
2	L–RL	− 0.10	0.45	[− 0.19, − 0.02]	− 2.44	.016	0.23	0.07	0.44	[− 0.01, 0.15]	1.72	.088	0.16
3	L–H	− 0.62	0.84	[− 0.98, − 0.26]	− 3.60	.002	0.73	0.73	0.86	[0.37, 1.01]	4.18	< .001	0.85
4	RL–L	− 0.04	0.52	[− 0.15, 0.06]	− 0.87	.387	0.09	0.01	0.49	[− 0.09, 0.10]	0.18	.857	0.02
5	RL–RL	− 0.40	0.48	[− 0.51, − 0.29]	− 7.20	< .001	0.84	0.48	0.54	[0.35, 0.60]	7.62	< .001	0.89
6	RL–H	− 0.60	0.60	[− 0.81, − 0.39]	− 5.81	< .001	1.00	0.70	0.57	[0.50, 0.90]	7.11	< .001	1.22
7	H–L	− 0.16	0.55	[− 0.42, 0.10]	− 1.29	.214	0.30	0.31	0.50	[0.06, 0.55]	2.64	.018	0.61
8	H–RL	− 0.62	0.56	[− 0.86, − 0.38]	− 5.41	< .001	1.10	0.94	0.70	[0.64, 1.24]	6.51	< .001	1.33
9	H–H	− 0.84	0.66	[− 1.12, − 0.55]	− 6.06	< .001	1.26	1.16	0.69	[0.86, 1.46]	8.29	< .001	1.73

*L* Low group, *R*L Relatively low group, *H* High group, *CI* confidence interval

**Table 8:** Results of one sample t-test for factor scores of two types of aggression at each profile

Profiles		Relational aggression						Overt aggression					
No.	Patterns	*M*	*SD*	95% CI	*t*	*p*	*d*	*M*	*SD*	95% CI	*t*	*p*	*d*
1	L–L	− 0.06	0.57	[− 0.10, − 0.02]	− 2.73	.007	0.11	− 0.04	0.62	[− 0.08, 0.01]	− 1.74	.081	0.07
2	L–RL	0.19	0.67	[0.07, 0.31]	3.06	.003	0.28	0.14	0.99	[− 0.04, 0.32]	1.50	.136	0.14
3	L–H	0.61	0.79	[0.28, 0.95]	3.83	< .001	0.78	0.43	1.24	[− 0.10, 0.95]	1.68	.101	0.34
4	RL–L	− 0.04	0.59	[− 0.16, 0.07]	− 0.77	.442	0.08	0.03	0.73	[− 0.11, 0.17]	0.42	.673	0.04
5	RL–RL	− 0.09	0.66	[− 0.24, 0.06]	− 1.16	.250	0.13	− 0.12	0.59	[− 0.26, 0.02]	− 1.74	.085	0.20
6	RL–H	0.44	0.72	[0.19, 0.69]	3.53	.001	0.61	0.27	1.06	[− 0.10, 0.65]	1.50	.143	0.26
7	H–L	0.10	0.75	[− 0.26, 0.46]	0.58	.571	0.13	− 0.08	0.40	[− 0.27, 0.11]	− 0.87	.395	0.20
8	H–RL	− 0.05	0.41	[− 0.22, 0.13]	− 0.57	.574	0.12	− 0.04	0.45	[− 0.23, 0.15]	− 0.42	.680	0.09
9	H–H	− 0.05	0.54	[− 0.28, 0.18]	− 0.45	.656	0.09	0.04	0.72	[− 0.27, 0.35]	0.26	.795	0.05

*L* Low group, *RL* Relatively low group, *H* High group, *CI* confidence interval

## Discussion

Theoretically, child loneliness is empathized as a subjective experience and is deeply related to difficulty with peers. This assumption means that poor interpersonal experiences are risk of loneliness. Meanwhile, simultaneously, this premise implies positive perspectives of interpersonal peer relations reducing loneliness. Our analyses investigating the interpersonal predictors of loneliness in late elementary school-aged children give the interim conclusion for the possibility regarding the effect of interpersonal experiences. In the following sections, we discuss the results of each analysis by a variable- and person- centered approach in turn, outlining their advantages.

The results of HLM revealed a negative correlation of positive peer relations with loneliness, while victimization and relational aggression were positively correlated with this dependent variable. Normally, these results would allow us to interpret that more positive peer relations as well as lower victimization and relational aggression reduce the risk of loneliness; however, these relations should be diligently interpreted because the results of the mover-stayer LTA did not fully uphold the interpretation. As shown, the factor scores of positive peer relations of decreaser (i.e. patterns 4, 7, and 8) were not significantly positive (rather negative) while the scores of victimization and relational aggression of decreaser were not significantly negative. If higher positive peer relations contribute to lower loneliness, their factor scores should be positive. Similarly, if lower victimization and relational aggression contribute to lower loneliness, their factor scores should be negative. Instead, the analysis supported only the inverse direction; that is, lower positive peer relations, higher victimization, and relational aggression were associated with higher loneliness. As revealed, the factor scores of positive peer relations of increaser (i.e. patterns 2, 3, and 6) were significantly negative; similarly, the factor scores of victimization and relational aggression of increaser were significantly positive except for pattern 2 for victimization (but positive with small effect size; Cohen’s *d* = 0.16). Overall, both approaches appear to support the predictors of only increased loneliness, not of decreased. As suggested by Kochenderfer-Ladd and Wardrop [16], these results caution us against over-interpretation by variable-centered approaches and indicate that a combination approach is a more useful and prudent approach to the data.

Based on the findings found in the result of within-level in HLM and mover-stayer LTA, the relation between victimization and loneliness at the cross-level interaction possibly indicates that a homeroom class characterized by higher victimization strengthens the relation in loneliness between T1 and T2. Generally, children with negative peer experiences tend to have distorted social cognitions [23] and are receptive to negative peer feedback [16] and social threat [36]. Classrooms with a higher score of victimization may evince continually rough or disrupted, which in turn might make children with higher loneliness become more sensitive and over-evaluate their lonely experience.

As with most of the previous work, more than half of our sample belongs to low stable pattern of loneliness (pattern 1: 61.67%); meanwhile, the percentage of individuals showing chronic loneliness (pattern 9: 2.11%) is small which may be comparable to or less than that in previous studies. However, focusing on combining the number of percentages from patterns 5, 6, 8, and 9, which are more than the relatively low level at both measurement points, approximately 15% of children are exposed to potential lonely experiences. Although some research did not report the increasing pattern of loneliness [11, 19], approximately 16% of children are categorized as increaser. Meanwhile, a trajectory characterized by decreasing loneliness in approximately 14% of our sample was found. These percentages were almost the same as those reported in previous studies. The mover-stayer LTA widely covers potential patterns of trajectories that previous research has found such as low levels of loneliness, decreasing, increasing, and chronically high patterns. We can observe the advantages of the mover-stayer LTA. Differentiation regarding trajectory patterns of loneliness among studies might give us potential perspectives on intervention for child loneliness through a comparison of school culture in each country where previous research was conducted. Moreover, we will have more practical tips for dealing with the problem of lonely children by focusing on the interpersonal experiences of children with decreasing patterns of loneliness.

### Practical implication

The present study showed risk factors causing higher loneliness: less-positive peer relations, higher victimization, and higher relational aggression; meanwhile, we could not find the interpersonal variables to reduce loneliness. These results show the importance of preventative practices in education. Namely, educators should focus on preventive practices for children’s loneliness rather than a therapeutic approach. Moreover, the cross-level interaction performed in HLM shows the significance of classroom effect: higher victimization at the class level strengthens an individual’s loneliness. Therefore, not only individual intervention such as teaching social skills for loneliness, but also coordinating interpersonal relationships between class members, resulting in boosting their school morale, would contribute to preventing children from future lonely experiences. Furthermore, Masi et al. [25] introduced the effects of the interventions to reduce loneliness and classified the four types: improving social skills, enhancing social support, increasing opportunities for social contact, and addressing maladaptive social cognition. Our finding regarding class level effect indicates the possibility that the effect of their interventions gets more strengthened in class- or group-based practice compared to the individual approach.

### Limitations

The present study has some limitations. First, we used subjective assessments of loneliness, positive peer relations, and victimization which likely led to inflated covariance. Additionally, although we used teachers’ assessments for the two types of aggression, teacher report is not the only prototype for such assessment. Therefore, it is suggested that studies in the future should test the robustness of the research findings by multiple assessments (e.g., observations and teachers’, parents’, and peers’ reports) to provide practical implications for vulnerable children for loneliness.

## Conclusion

From a variable-centered approach, this study identified the following variables as interpersonal predictors of decreasing future loneliness: more positive peer relations, lower victimization, and lower relational aggression; however, a person-centered approach could not support the findings. Conversely, the person-centered approach indicated findings in the inverse direction in which less positive peer relations, higher victimization, and higher relational aggression are predictors of higher future loneliness. In other words, we should not interpret the relation in the desired direction without deep consideration. Overall, using a combination of variable- and person-centered approaches provides insightful contributions and sounds an alarm about overreliance on the variable-centered approach for children’s study. For practical suggestion, in order to protect young children from loneliness, we conclude that it will be more beneficial to prevent the development of loneliness rather than to apply interventions to reduce loneliness once established.

## Supplementary Information

**Additional file 1: Appendix 1. **Syntax for mover-stayer LTA in Mplus.

## Data Availability

The datasets used and/or analyzed during the current study are available anytime and can be obtained from the corresponding author on reasonable request.
