# Supplementary material for: Interpersonal predictors of loneliness in Japanese children: variable- and person-centered approaches
Source: BMC Psychol. 2022 Jun 29;10:164. doi: 10.1186/s40359-022-00853-1 (PMC9245278; doi:10.1186/s40359-022-00853-1)
Supplement: Supplementary file 1 [file 40359_2022_853_MOESM1_ESM.docx]

Appendix 1

Syntax for mover-stayer LTA in Mplus

```
TITLE:  mover-stayer LTA for loneliness
DATA:
      FILE IS "C:\Users\......\...txt";
VARIABLE:
      NAMES =  l1 l2 l3 l4 l5 m1 m2 m3 m4 m5;
      USEVARIABLES =  l1 l2 l3 l4 l5 m1 m2 m3 m4 m5;
      MISSING = .;
      CLASSES = c(2) c1(3) c2(3);
ANALYSIS:
      TYPE = MIXTURE;
      PARAMETERIZATION = PROBABILITY;
      STARTS = 100 20;
      PROCESSORS = 8;
Model:    %OVERALL%
             c1 on c;
Model c:  %c#1%
             c2 on c1;
             %c#2%
             c2#1 on c1#1@1; c2#2 on c1#1@0;
             c2#1 on c1#2@0; c2#2 on c1#2@1;
             c2#1 on c1#3@0; c2#2 on c1#3@0;
Model c1: %c1#1%
             [l1] (1);  [l2] (2);  [l3] (3);  [l4] (4);  [l5] (5);
             %c1#2%
             [l1] (6);  [l2] (7);  [l3] (8);  [l4] (9);  [l5] (10);
             %c1#3%
             [l1] (11);  [l2] (12);  [l3] (13);  [l4] (14);  [l5] (15);
Model c2: %c2#1%
             [m1] (1);  [m2] (2);   [m3] (3);  [m4] (4);  [m5] (5);
              %c2#2%
             [m1] (6);  [m2] (7);  [m3] (8);   [m4] (9);  [m5] (10);
             %c2#3%
             [m1] (11);  [m2] (12);  [m3] (13);   [m4] (14);   [m5] (15);
OUTPUT:  STDYX CINTERVAL TECH1 TECH8 TECH15;
```

Note. Each item for loneliness is correspondent to l1 ~ l5 (Time 0) and m1 ~ m5 (Time 2)
